# *Pogonias courbina* sperm characteristcs in its first reproductive season

**DOI:** 10.7717/peerj.15600

**Published:** 2023-07-18

**Authors:** Jhony Lisboa Benato, Danilo Streit Jr, Nathalia Dos Santos Teixeira, Rômulo Batista Rodrigues, Thaiza Rodrigues de Freitas, Marcelo Okamoto, Ricardo Rodrigues, Raquel Santos dos Santos, Renata Villar Dantas, Ana Paula de Abreu Balbinot, Rodrigo Ribeiro Bezerra de Oliveira, Lucas Campos Maltez, Olivia Menossi, Luis Andre Sampaio

**Affiliations:** 1 Animal Science Research Program, Universidade Federal do Rio Grande do Sul, Porto Alegre, RS, Brasil; 2 Veterinary Research Program, Universidade Federal do Rio Grande do Sul, Porto Alegre, RS, Brasil; 3 Department of Animal Science and Biological Sciences, Universidade Federal de Santa Maria, Santa Maria, RS, Brazil; 4 Laboratory of Marine Fish Culture, Oceanographic Institute, Universidade Federal do Rio Grande, Rio Grande, RS, Brasil

**Keywords:** Sperm abnormalities, Sperm morphometry, Sperm kinetics, Artifical reproduction, Southern black drum

## Abstract

Southern black drum (*Pogonias courbina*) is a species distributed along the western Atlantic Ocean, and it is the largest Sciaenidae observed in the coast of Rio Grande do Sul state, Brazil. However, it is listed as a vulnerable species at The IUCN Red List of Threatened Species™, and their fishing is prohibited. The objective of this study was to determine the sperm characteristics of *P. courbina*. Sperm samples of five young males (two-year-old fish) were collected through abdominal pressure. The sperm kinetics parameters were sperm motility (MOT) 10.7 ± 5.6%, curvilinear velocity (VCL) 120.07 ± 16.16 mm s ± 1, average path velocity (VAP) 75.64 ± 23.78 mm s ± 1, straight-line velocity (VSL) 62.49 ± 15.83 mm s ± 1, straightness (STR) 83.9 ± 5.3%, wobble (WOB) 61.9 ± 12.7%, beat cross frequency (BCF) 42.981 ± 4.627 Hz and progression (PRG) 1,805.4 ± 564.5 µm. The proportion of normal spermatozoa was 35.6 ± 6.1%. About the abnormalities observed, 22.7% occurred in the tail (short tail = 0.6 ± 0.5%, distally curled tail = 2.4 ± 1.6%, strongly curled tail = 1.9 ± 1.3%, broken tail = 7.9 ± 5.1%, folded tail = 5.5 ± 0.8%, loose tail = 4.4 ± 1.9%); 14.2% occurred in the head (degenerate head = 4.2 ± 1.6%, microcephaly = 1.8 ± 2.5%, loose head = 8.2 ± 2.1%) and 27.5% of the spermatozoa showed cytoplasmatic gouts (proximal gout = 20.0 ± 8.4%, distal gout = 7.5 ± 2.8%). Besides that, a correlation analysis was performed between sperm morphology and kinetics parameters, and the spermatozoa were measured for the morphometric parameters. There was a positive correlation between BCF and normal spermatozoa (*r* =  0.9269). A negative correlation occurred between BCF and loose head (*r* =  −0.9047); WOB and strongly curled tail (*r* =  −0.8911); and PROG and strongly curled tail (*r* =  −0.9191). The morphometric measures found for the head were length of 2.50 ± 0.21 µm and width of 2.12 ± 0.22 µm, and for the tail it was length of 37.97 ± 2.01 µm. It was possible to verify that the animals have sperm characteristics that indicate reproductive aptitude, but an abnormal behavior on sperm activation and high presence of the cytoplasmic gout abnormality indicates that the animals are not fully mature in their first reproductive season. This work contributes to a better understanding of the *P. courbina* spermatic parameters, what can be allies to recovery this species population in nature and promote its production in fish farms.

## Introduction

The Southern black drum *Pogonias courbina* (Lacepede, 1803) is a demersal coastal fish species distributed along the western Atlantic Ocean, including Brazil, Uruguay and Northern Argentina. This species has recently been redescribed based on its geographic isolation, on morphological and molecular difference with populations of black drum (*Pogonias cromis*) from the North Atlantic (Azpelicueta et al., 2019). This species inhabits estuaries, bays and coastal waters, tolerating a wide range of salinities and temperatures and it is the largest sciaenid observed in the coast of Rio Grande do Sul state, Brazil (Dos Santos & Velasco, 2021). The first maturation is accomplished with approximately 2 years of life, it lives up to 55 years and reaches over 60 kg and 1,400 mm total length (Machado et al., 2020; Dos Santos & Velasco, 2021).

Adult fish have the behavior of forming large aggregations during the spawning season, which occurs between October and March (Urteaga & Perrotta, 2001; Haimovici & Cardoso, 2016). Females have high fertility rates spawning multiple times over the season, with a larval phase of about 4 months (Machado et al., 2020). However, since the 1980 decade, the Southern black drum has been a significant decline in its landings (IBAMA/CEPRG, 2011), and in 2016 was considered collapsed (Haimovici & Cardoso, 2016). This fact is mainly due to its overfishing and the characteristic of forming large aggregations during the reproductive period. Furthermore, the species has been recently added to the Brazilian list of endangered species (MMA, 2022) as endangered (EN), and considered vulnerable (VU) by The IUCN Red List of Threatened Species (Haimovici et al., 2020). Therefore, its fishing is prohibited in Brazil.

In efforts to re-establish the population of the Southern black drum in the wild, we can reproduce this species in laboratory and stimulate stocking enhancement programs. This methodology already has been implemented worldwide for species such as Siberian sturgeon (Gebauer et al., 2021), Atlantic salmon (Jokikokko et al., 2006; Czerniawski, Pilecka-Rapacz & Domagala, 2011), Japanese flounder (Tomiyama et al., 2011), and sea trout parr (Krepski & Czerniawski, 2019). For the success of these programs and for artificial reproduction, it is important to know the characteristics of the animals’ gametes and to be able to assess their quality during reproduction. A few studies have been conducted for *P. courbina* in nature, where the age for onset of reproductive maturity was determined (Dos Santos & Velasco, 2021), along with the description of reproductive parameters of females during the breeding season (Macchi, Acha & Lasta, 2002). The genetic connectivity between populations of this species was also studied (Machado et al., 2020). However, there are no studies evaluating the sperm characteristics for this species. Besides that, several authors have already verified a high correlation between sperm characteristics and fertilization success (Gallego et al., 2013; Beirão et al., 2015; Neumann, Sanches & Bombardelli, 2019). In addition, the recent study by Ito et al. (2022) show that the fertilization modes of marine fish and their reproductive strategy are closely related to sperm morphology, morphometry and motility, which makes knowledge of sperm characteristics essential for reproductive success in captivity (López-Hernández et al., 2018).

Therefore, the objective of this study was to evaluate the morphological, morphometric, and kinetic characteristics of spermatozoa from *P. courbina* raised from birth in captivity. With this knowledge, it will be possible to evaluate its viability for use in artificial reproduction programs, stock enhancement programs, and the formation of a germplasm bank of the species.

## Materials & Methods

### Ethics statement

All experimental procedures imposed on animals were approved by the Animal Use Ethics Committee of Federal University of Rio Grande (CEUA–FURG) (project 23116.006908/2017—81), and all procedures used were consistent with the established guidelines of the National Council for the Control of Animal Experimentation (CONCEA).

### Animal care

Two-year-old black drum males (*Pogonias courbina*) were used in this study (38.3 ±  1.8 cm). The animals originated through semi-natural reproduction from wild captured broodstock. The animals have been raised in captivity since hatching, with food based on natural food (chopped fish and squid purchased from local fish markets). A total of 45 animals (males and females) were kept in 16,000 L tanks placed in a recirculating aquaculture system, which uses natural seawater (temperature = 26.49 ± 0.59 °C, pH = 7.69 ±  0.11, dissolved oxygen = 5.3 ±  0.62, salinity = 35.9 ±  1.35 g.L^−1^).

### Hormonal induction

During the reproductive season in December 2021, five males (1.14 ±  0.14 kg) were carefully removed from the maintenance tank and placed individually in a 100 L tank containing eugenol at a concentration of 50 mg/L until they reached a state of deep anesthesia, based on the fish anesthesia stage classifications of Vidal et al. (2007). It is achieved when the fish loses muscle tone as well as balance, having a slow but regular opercular movement. After being anesthetized, the weight and size of the animals were measured to define the dose of hormone to be used for hormone induction. The animals were induced using crude carp pituitary extract at a dose of 5 mg/kg, they were then placed in 1,000 L tanks.

### Sperm collection

After a thermal accumulation of 240 degree-hours (10 h, 24 °C), the animals were removed from the tanks for sperm collection. An anteroposterior massage was applied in the abdominal region with the fish slightly inclined with the head upwards, collecting the sperm with a 3 mL syringe. During collection, the initial sperm obtained was discarded, and possible contamination with feces, blood, or urine was avoided. After the collection, samples were immediately diluted in HBSS extender (pH 7.50 and osmolarity of 281 mOsm/L, adapted from Wayman, Thomas & Tiersch (1997)) at a final proportion of 1:10. The animals were immobilized with wet towels, and their eyes covered to facilitate handling and avoid possible injuries to the individual handling the fish. The sperm samples were transferred to 1.5 mL microtubes and then evaluated for sperm pre-activation using an optical microscope (Nikon Eclipse E200; Nikon, Tokyo, Japan) at 100x magnification. The samples were kept in a closed styrofoam box containing chemical ice, at an approximate temperature of 10 °C for a maximum time of 1 h until analysis.

### Sperm kinetic parameters

For the evaluation of sperm kinetics, the sperm was diluted in HBSS solution (pH 7.50 and osmolarity of 325 mOsm/L) at 1:9 µL ratio and then activated in HBSS solution (pH 7.50 and osmolarity of 600 mOsm/L) in a ratio of 1:99 µL. Immediately after activation, 5 µL of this solution were placed in a Neubauer hemocytometer chamber, covered with a coverslip. The chamber was placed under a transmitted light microscope (Nikon Eclipse E200; Nikon, Tokyo, Japan), connected to a high velocity camera (Basler AC640–120uc, 120 fps, Ahrensburg, Germany), at 100x magnification. After that, five videos of each fish were recorded, using Pylon Viewer 4 software (Version 4.1.0.3660 64-Bit; Basler, Ahrensburg, Germany) at a capture rate of 100 frames per second, in 1,440 × 1,080 pixels resolution. The duration of the recorded videos was 20 s, and the analysis was performed in a period of 0.5 s (50 frames), 10 s after activation. The videos were edited using the VirtualDub software and analyzed using the ImageJ software, through CASA plugin.

Several tests were carried out to define the parameters used in the CASA analysis, until the settings for *a* = 5, *b* = 40, *c* = 50, *d* = 8 e =4, *f* = 6, *g* = 10, *h* = 5, *i* = 1, *j* = 4, *k* = 6, *l* = 35, *m* = 80, *n* = 80, *o* = 50, *p* = 60, *q* = 100, *r* = 431,03, *s* = 0, *t* = 0 (adapted from Neumann, Sanches & Bombardelli, 2019) were defined. The parameters evaluated were sperm motility (MOT, %), curvilinear velocity (VCL, µm s^−1^), the real speed of the trajectory of the spermatozoa, average path velocity (VAP, µm s^−1^), the average speed of the spermatozoa, straight-line velocity (VSL, µm s^−1^), the average speed between the final and initial point of movement, straightness (STR, %), relates how close the cell’s trajectory is to a straight line, wobble (WOB, %), oscillation index, progression (PROG, µm), total distance traveled during the analysis, and beat cross frequency (BCF, Hz), number of times the sperm head crosses the axis of motion (Matos et al., 2008).

### Spermatozoa morphology

At the time of sperm collection, an aliquot of 1 µL was collected and fixed in 249 µL s of a 10% formalin buffered saline solution. To prepare 1 L of this solution, 6.5 g of anhydrous dibasic sodium phosphate (Na_2_HPO_4_) were dilute in 450 mL of distilled water, 4.0 g of monobasic sodium phosphate monohydrate (*NaH*_2_*PO*_4_*.H*_2_*O)* were dilute in another 450 mL of distilled water, the two solutions were mixed and 100 mL of formaldehyde (37–41%) was added.

The analysis of sperm morphology was performed using rose bengal staining (Streit Jr et al., 2004). To prepare the dye, 3 g of powdered rose bengal were diluted in 50 mL of distilled water. After dissolution, 1 mL of commercial formaldehyde (37 to 41%) was added and the volume was completed with distilled water to 100 mL. Then, 30 µL of sperm from each fish were mixed with 1 µL of rose bengal dye solution. The microtube content was manually homogenized. With a pipette, a volume of 10 µL of the final solution was deposited on a histological slide. The slide was positioned at an angle of 45°  with the surface, and the deposited drops flowed to the edge. After this step, the slides were dried at an ambient temperature of 25 °C.

**Figure 1:**
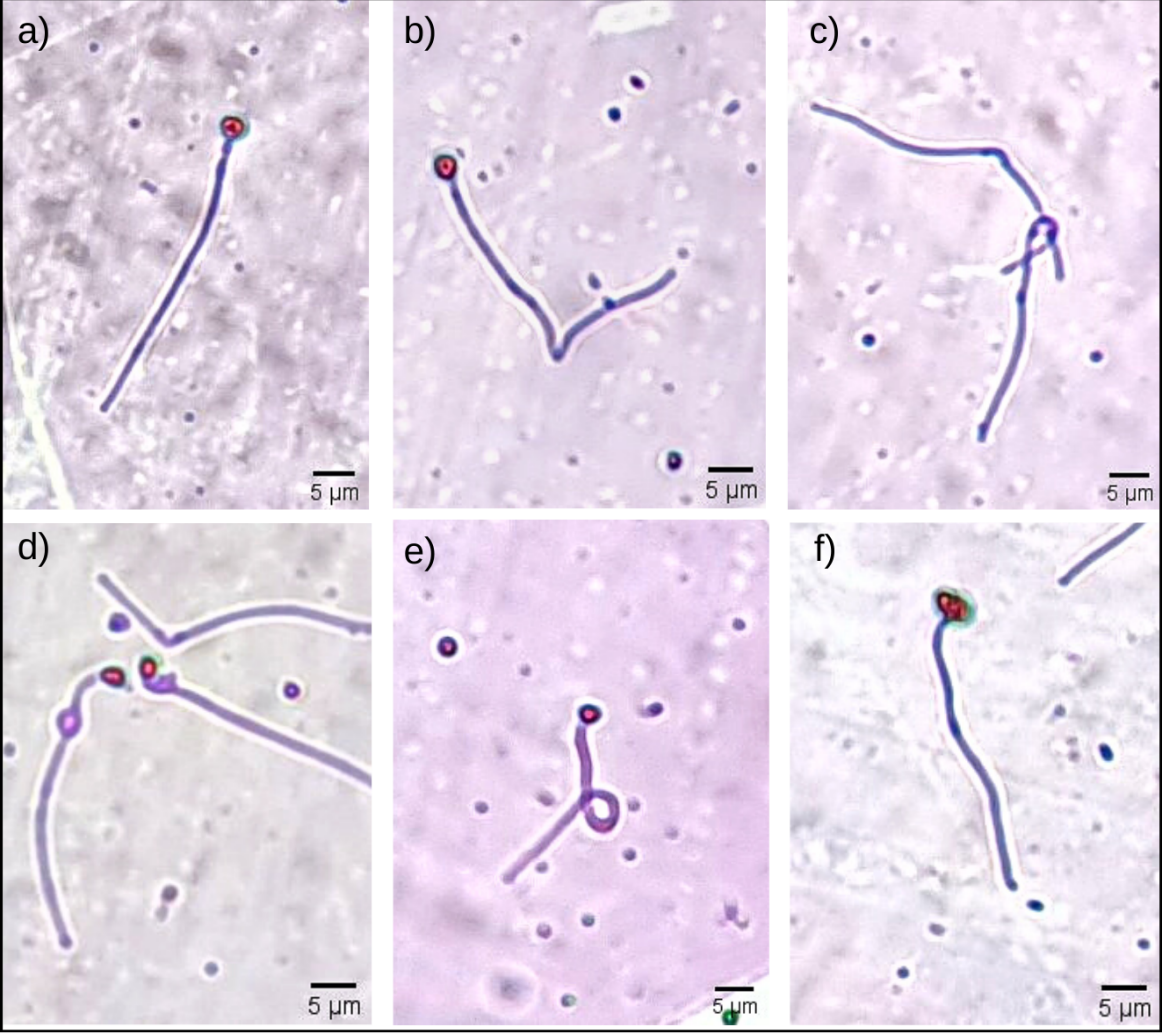
Some morphological abnormalities of *Pogonias courbina* sperm. (A) Normal sperm, (B) bent tail, (C) loose tail, (D) distal gout, (E) coiled tail, (F) degenerate head. Magnification level 5 µm.

Sperm morphological changes were classified into head abnormalities (isolated head, degenerated, macrocephaly, microcephaly) and tail abnormalities (broken tail, coiled tail, strongly coiled tail, short tail, bent tail, proximal and distal cytoplasmic gouts) (Herman, 1994). With the slides ready, the analysis was performed by observing 200 spermatozoa from each male in a transmitted light microscope (Nikon Eclipse E200; Nikon, Tokyo, Japan), at 400x magnification, and the classification of morphological abnormalities was obtained subjectively, adapted from Da Costa et al. (2022). In Fig. 1, it is possible to observe some of the sperm abnormalities.

### Spermatozoa morphometry

For the spermatozoa morphometry analysis, histological slides were prepared with rose bengal staining, as the same way to analyze sperm morphology. Then, the slides were dried at room temperature (25 °C) and observed under a transmitted light microscope (Nikon Eclipse E200; Nikon, Tokyo, Japan).

**Figure 2:**
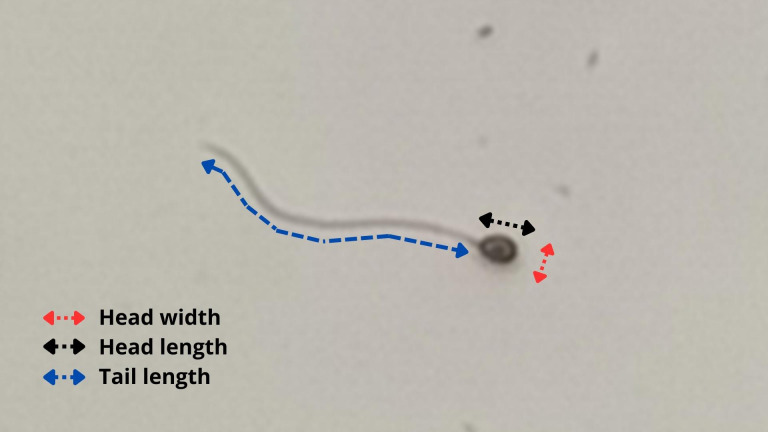
Methodology used to evaluate sperm morphometry in *P. courbina*. Head width (in red); head length (in black); tail length (in blue).

A Basler camera (Basler AC640–120uc, 120 fps, Ahrensburg, Germany) was used to photograph the spermatozoa at 400x magnification, using defined settings for better observation of them. Then, a histological measurement slide was photographed under the same microscope and camera settings to serve as a scale. After that, the spermatozoa had head length, head width and tail length measured using the software ImageJ (103 cells of three animals). Figure 2 shows a representation of the methodology used to evaluate sperm morphometry.

### Membrane integrity

Sperm membrane integrity was assessed by the double - staining LIVE/DEAD™ Sperm Viability Kit (Thermo Fisher, Waltham, MA, USA), which is composed of the fluorescent dyes SYBR-14 (for live cell nucleic acid staining) and propidium iodide (PI, for dead cell nucleic acid staining that penetrates through damaged plasma membrane), adapted from the method proposed by Hagedorn et al. (2009), with modifications. The sperm (20 µL) was mixed with 0.25 µL SYBR-14 (0.02 mM) and incubated in the dark at room temperature (25 °C) for 4 min. After that, 1.0 µL PI (1.19 mM) was added to the sample, which was incubated for 1 min under the same conditions. After this incubation, the sperm was used to prepare histological slides and analyzed under fluorescence microscope (Carl Zeiss, Jena, Germany, 400 × magnification, Axioplan-Zeiss epifluorescence microscope). Three slides per animal were evaluated, with a total of 200 spermatozoa per slide, and after determining the number of spermatozoa with intact membranes and with ruptured membranes, the membrane integrity rate was calculated.

### Statistical analysis

An exploratory analysis was performed because of the scarcity of information on the species herein analyzed. First, a descriptive statistical analysis of the data was performed for selecting relevant data regarding sperm morphology, morphometry, membrane integrity and sperm kinetics parameters (CASA). Regarding descriptive statistics, the following data were calculated: number of values (n), minimum, maximum, median, percentile (25%), percentile (75%), mean, standard deviation, standard error of mean and coefficient of variation.

To assess the most prevalent sperm abnormality for each specimen, the abnormality data were subjected to normality analyses (Shapiro Wilk, Kolmogorov Smirnov, or D’Agostino-Pearson) and Levene’s homogeneity test. After confirming compliance with the statistical assumptions, the data were submitted to one-way analysis of variance (ANOVA), followed by Tukey’s test or Kruskal–Wallis test, followed by Dunn’s test. To verify the correlation between the values of sperm morphology and the sperm kinetics parameters, Pearson’s correlation analysis was performed. The analysis and graphics were carried out using the GraphPad Prism 9.0 software.

## Results

### Characterization of sperm kinetic parameters

The results of the descriptive statistic of sperm kinetic parameters can be observed in Table 1.

### Frequency and type of sperm abnormalities

The results of the descriptive statistic of morphological analysis can be observed in Table 2.

**Figure 3:**
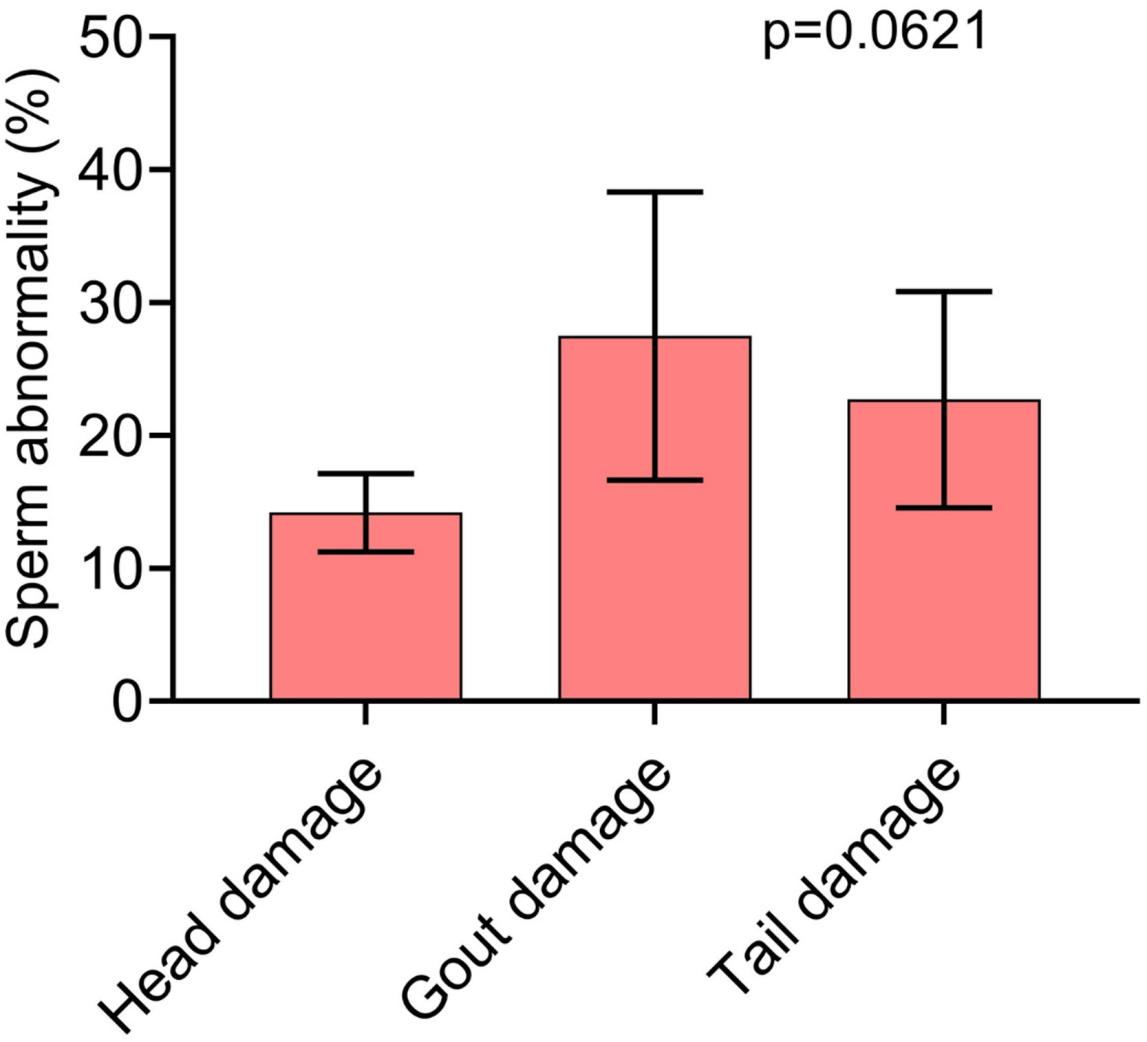
Pearson correlation between sperm morphology and kinetics variables with significant effect (*p* < 0.05).

In Fig. 3, we can see which were the most prevalent sperm abnormalities. In Fig. 3A, it is possible to observe that the abnormality of proximal gout occurred more frequently than other abnormalities, such as macrocephaly, microcephaly, short and strongly coiled tail. However, in Fig. 3B, it is possible to observe that there was no statistical difference between the head and tail abnormalities and the occurrence of cytoplasmic gouts.

**Table 1:** Descriptive statistics of sperm kinetics (CASA) parameters.

	MOT	VCL	VAP	VSL	STR	WOB	PROG	BCF	SPTz
Number of values	5	5	5	5	5	5	5	5	5
Minimum	3.3	98.6	50.7	44.9	76.3	46.3	1051	38.3	196.0
25% Percentile	4.9	107.2	52.2	46.4	78.9	48.9	1323	39.2	205.0
Median	12.5	118.4	76.6	64.4	84.1	64.7	1649	40.6	341.0
75% Percentile	15.5	133.8	98.7	77.6	88.9	73.8	2366	47.9	433.0
Maximum	17.3	143.5	105.4	80.5	89.2	74.1	2424	48.7	483.0
Range	14	44.8	54.7	35.5	12.9	27.8	1373	10.4	287.0
Mean	10.7	120.1	75.6	62.5	83.9	61.9	1805	42.9	323.4
Std. Deviation	5.6	16.2	23.8	15.8	5.4	12.7	564.5	4.6	119.9
Std. Error of Mean	2.5	7.2	10.6	7.1	2.4	5.7	252.4	2.1	53.6
Coefficient of variation	52.8	13.5	31.4	25.3	6.4	20.5	31.3	10.8	37.1

**Notes.**

Sperm kinetics (CASA)

MOT sperm motility (%) VCL curvilinear velocity (µm s^−1^) VAP average path velocity (µm s^−1^) VSL straight-line velocity (µm s^−1^) STR straightness (%) WOB wobble (%) PROG progression (µm) BCF beat cross frequency (Hz) SPTz number of spermatozoa (n)

**Table 2:** Descriptive statistics of normal and abnormal sperm parameters.

**Descriptive statistics**	NS	DH	MAC	MIC	LH	PG	DG	LT	ST	BT	FT	DCT	SCT
Number of values	5	5	5	5	5	5	5	5	5	5	5	5	5
Minimum	29.0	2.0	0.0	0.0	5.5	11.0	4.5	1.5	0.0	3.5	4.5	0.5	0.0
25% Percentile	30.5	2.5	0.0	0.0	6.2	12.2	5.5	2.7	0.0	3.7	4.7	1.2	0.5
Median	33.0	5.0	0.0	0.0	8.0	21.0	6.5	4.5	1.0	6.5	5.5	2.0	2.5
75% Percentile	42.0	5.5	0.0	4.5	10.2	27.2	10.0	6.0	1.1	12.7	6.2	3.7	3.0
Maximum	43.5	5.5	0.0	5.0	10.5	32.5	12.0	6.0	1.1	16.0	6.5	5.0	3.0
Mean	35.6	4.2	0.0	1.8	8.2	20.0	7.5	4.4	0.6	7.9	5.5	2.4	1.9
Std. Deviation	6.1	1.6	0.0	2.5	2.1	8.4	2.8	1.8	0.6	5.1	0.8	1.6	1.3
Std. Error of Mean	2.7	0.7	0.0	1.1	0.9	3.8	1.3	0.8	0.2	2.3	0.3	0.7	0.6
Coefficient of variation (%)	17.2	38.2	–	138.3	25.4	42.2	37.4	42.1	91.5	64.8	14.4	68.1	70.6

**Notes.**

Sperm morphology parameters (%)

NS Normal spermatozoa DH Degenerate head MAC Macrocephaly MIC Microcephaly LH Loose head PG Proximal gout DG Distal gout LT Loose tail ST Short tail BT Broken tail FT Folded tail DCT Distally curled tail SCT Strongly curled tail

### Relationship between kinetic parameters and sperm morphology

**Figure 4:**
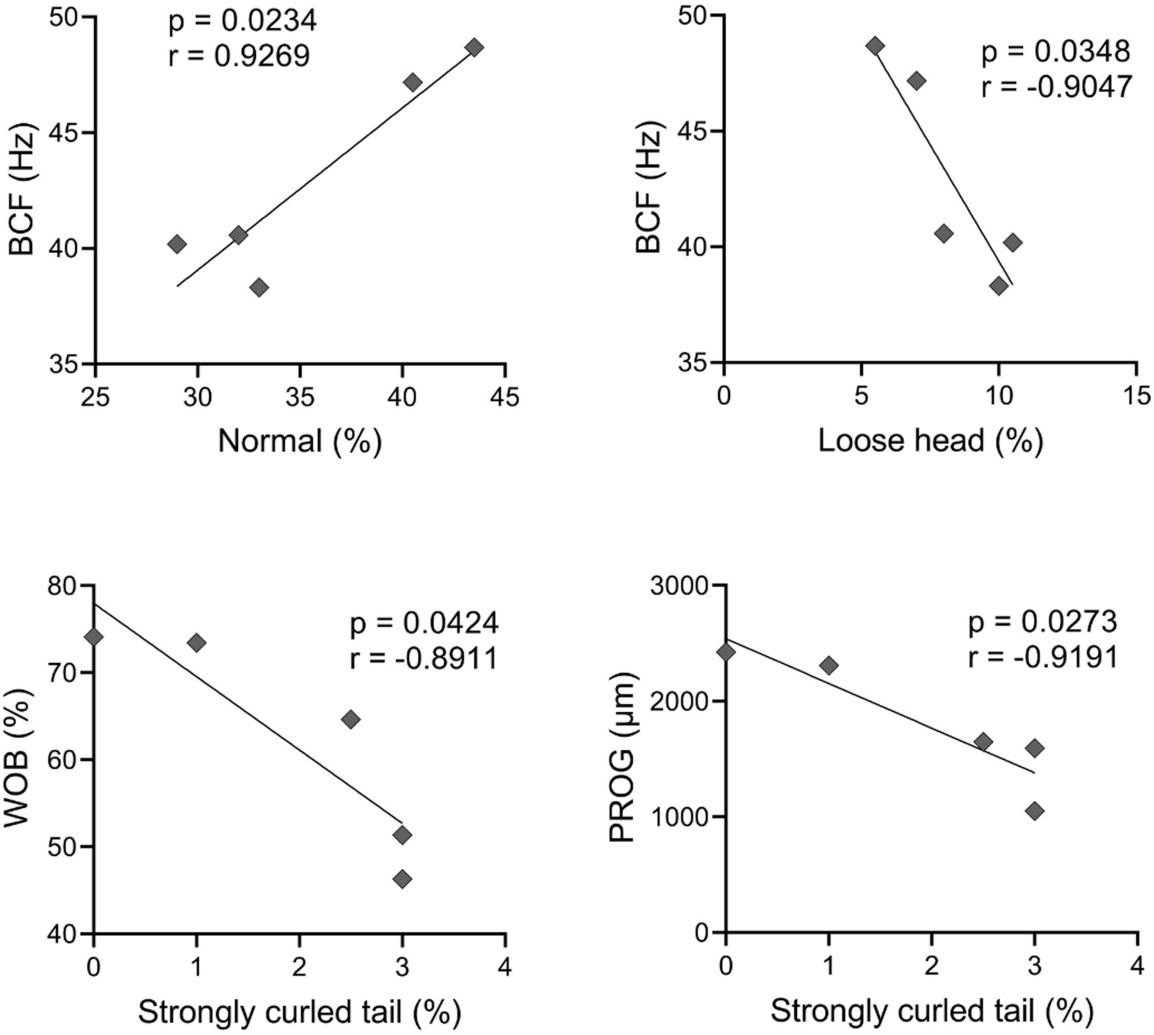
Pearson correlation between sperm morphology and kinetics variables with significant effect (*p* < 0.05).

A Pearson correlation analysis was conducted, and it was found significant effect between some abnormalities and kinetics parameters, as observed in Fig. 4. There was a positive correlation between BCF and normal spermatozoa (*r* = 0.9269); and BCF and microcephaly (*r* = 0.9497). A negative correlation occurred between VAP and microcephaly (*r* = −0.8991); VSL and microcephaly (*r* = −0.9285); WOB and microcephaly (*r* = −0.9256); BCF and loose head (*r* = −0.9047); WOB and strongly curled tail (*r* = −0.8911); and PROG and strongly curled tail (*r* = −0.9191).

### Spermatozoa morphometry

**Figure 5:**
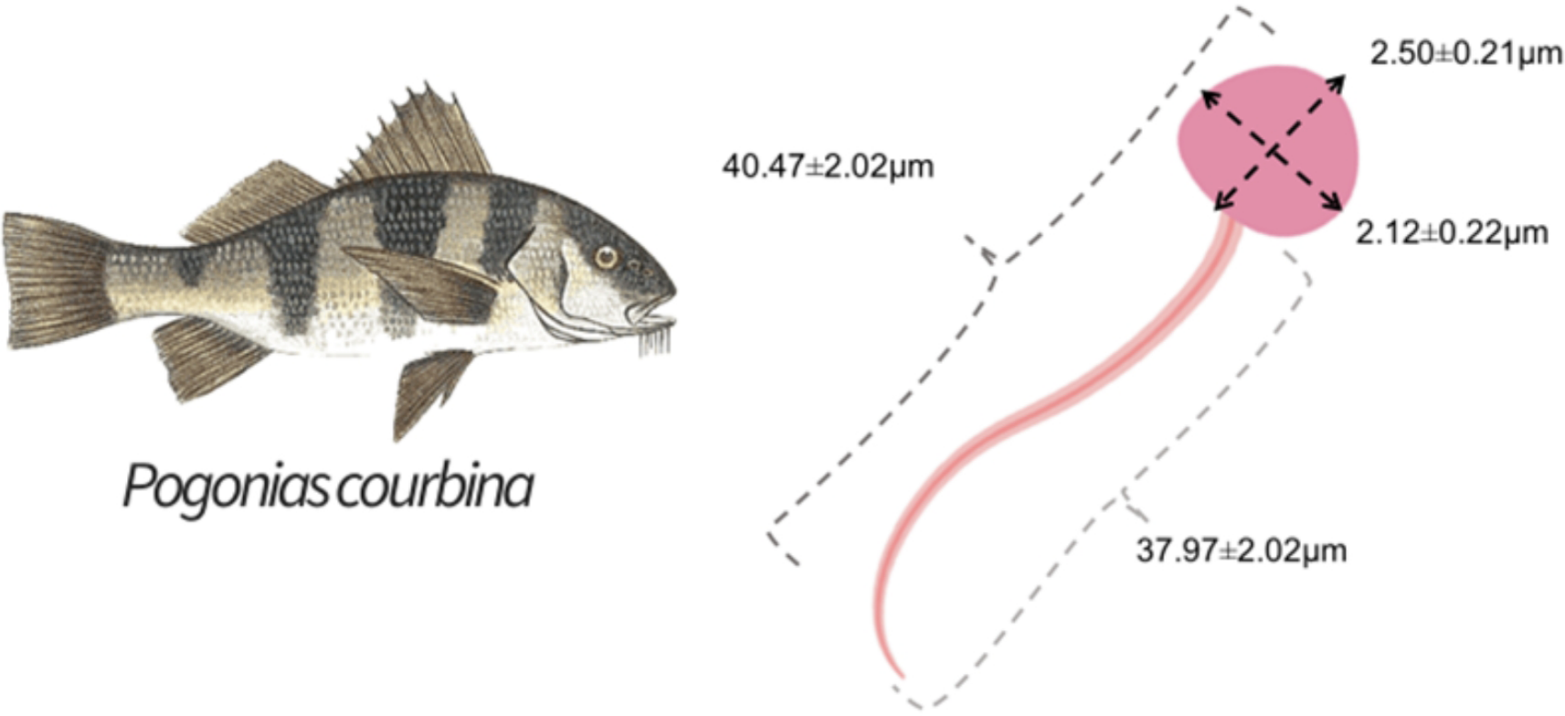
Measurement of sperm morphometry with rose bengal dye and ImageJ software.

The mean measures found for the head were length of 2.50 ±  0.21 µm and width of 2.12 ± 0.22 µm, and for the tail it was length of 37.97 ± 2.01 µm, with a mean total length of 40.47 ± 2.22 µm. Figure 5 shows the results of sperm morphometry measurements.

### Membrane integrity

The results of the descriptive statistic of membrane integrity can be observed in Table 3. The membrane integrity percentage of spermatozoa stained by LIVE/DEAD assay was 71.47 ± 15.41%.

## Discussion

In an effort to build a laboratory reproduction protocol for the Southern black drum (*P. courbina*), listed as an endangered species, this unprecedented study brings important revelations. By approaching the microscopic reproductive aspects of *P. courbina*, the findings consolidate the information that was observed regarding the macroscopic reproductive aspects of the species. The presence of a high concentration of cytoplasmic gout in sperm becomes a crucial finding to determine the reproductive maturity and, consequently, the recommendations for a future reproductive protocol in the laboratory for this species (Da Costa et al., 2022).

The analysis of sperm kinetic parameters is used to compare treatments and verify sperm quality (Gallego & Asturiano, 2018; França et al., 2022). In the first activations, an abnormal sperm behavior was observed, when compared to other species that perform external fertilization. After activation, fields with a high concentration of high-speed active spermatozoa were observed, while nearby fields had inactive or very low-speed active spermatozoa. In some fields of observation, the motility rate obtained, as subjective way, was close to 100%, however, in other fields of the slide the activation occurred progressively over time and the spermatozoa had a low velocity, with a motility rate close to 10%. To try to solve this problem, the sperm was diluted in the HBSS extender solution (325 mOsm/L, pH 7.5) in a ratio of 1:9 (Abinawanto, Yimastria & Pertiwi, 2018). Additionally, activation was performed using tank water and other proportions of sperm and activator, but no change was observed in sperm movement speed and in motility pattern. In a recent study, (Da Costa et al., 2019) found a high correlation (*r* = 0.91) between the percentage of normal sperm and motility. These results may explain the abnormal behavior of the sperm motility observed in the present study. Another hypothesis is that this phenomenon occurs due to characteristic of the *P. courbina* sperm, and the behavior we observed included constant sperm activation, even with sperm diluted after collection and without the addition of activator. This leads us to believe that some other factor influences sperm activation, which occurs gradually and unevenly, and the sperm can be maintained a for up to more than one day after collection. We also believe that it can be an intrinsic characteristic of the species or individuals assessed, because the analysis was repeated several times and with different sperm:activator ratios and the sperm movement pattern was the same. Further analyzes should be conducted to elucidate this behavior, such as testing other activating solutions.

**Table 3:** Descriptive statistics of membrane integrity. Results for membrane integrity by LIVE-DEAD assay.

**Descriptive statistics**	Live/Dead (%)
Number of values	5
Minimum	59.3
25% Percentile	59.4
Median	64.5
75% Percentile	87.1
Maximum	94.2
Mean	71.5
Std. Deviation	15.2
Std. Error of Mean	6.8
Coefficient of variation (%)	21.3

With the analysis of sperm morphology, it was found that 64.4% of spermatozoa had abnormalities. A high frequency of sperm abnormalities can be detrimental to reproduction and drastically decrease fertilization success due to sperm not reaching the oocyte (Da Costa et al., 2022). These abnormalities can be caused by many factors, including genetic factors, disease, stress, temperature, test substances, and spermatogenesis problems (Billard et al., 1995). Abnormalities of the sperm head can represent a problem since each species of fish has very specific characteristics in the micropyle of the oocytes that allow the passage of sperm. Changes in the size or shape of the head can compromise fertilization success (Pšenička et al., 2008). Tail abnormalities, on the other hand, alter cell displacement and kinetics parameters. Therefore, sperm with tail abnormalities have less success in reproduction due to changes in their oscillation and movement pattern, resulting in a decrease in their progressivity towards the oocytes. In *P. courbina*, the most frequently observed sperm abnormality was the presence of cytoplasmic gout, with a rate of 27.5%. As determined by Dos Santos & Velasco (2021), the age of the first reproductive maturation of fish occurs at approximately 2.13 years of age. Although, the authors stated that even if they can reproduce at this age, the fish will be 100% mature after 4 years. The animals used in this experiment were approximately two years old, so, they were reaching reproductive maturity, which is consistent with the results observed in the morphological analysis.

It has already been verified by several authors (Gallego et al., 2013; Beirão et al., 2015; Neumann, Sanches & Bombardelli, 2019), that there is a high correlation between high speed sperm movement and fertilization rates. In addition to speed, factors such as motility rate, progressive motility, straightness, and wobble also show a high correlation with the fertilization rate, as observed by Gallego et al. (2013) in pufferfish. Besides that, morphological parameters determine the characteristics of sperm motility after activation (Emel’yanova & Pavlov, 2020). With the correlation analysis, it was possible to verify the influence of certain abnormalities on sperm kinetics. The variables normal sperm and BCF showed a positive correlation (*r* = 0.927). This means that increased sperm abnormalities can decrease sperm velocity, in addition to causing changes in other kinetic parameters. Other abnormalities, such as a heavily curled tail, showed a negative correlation with parameters such as WOB (*r* = −0.891) and PROG (*r* = −0.919), indicating that not only head abnormalities, but also tail abnormalities can impair sperm movement and impair their reproductive efficiency. As there are no studies evaluating the sperm kinetics of *P. courbina*, it was decided to compare parameters such as VCL and RET with the meagre, *Argyrosomus regius*, another marine fish species of the Scianidae family. The values found in present study for *P. courbina* were 120 µm/s for VCL, while in the study of Santos et al. (2018), VCL was 140 µm/s in meagre sperm. For straightness (RET) analysis, the observed for meagre was 56%, while in Southern black drum in the present study the value was 83,9%. This suggests that the pattern of spermatozoa movement and possibly the reproductive strategy of these two species is different. Moreover, it is possible that the *P. courbina* spermatozoa movement pattern was compromised by other factors, such as the high frequency of observed sperm abnormalities (Da Costa et al., 2022).

There is no information on sperm morphometry of *P. courbina*, therefore, the study of Gage et al. (2002) was selected to compare this parameter. As salmon is a well-studied species, with an established production model, it can serve as a comparison and it has a wide range of information about its reproductive parameters. The mean sperm morphometric measures found was 40.47 ± 2.22 µm, close to the results found by Gage et al. (2002), who performed sperm morphometry in *Salmo salar* and observed variations in the animals, with values of total sperm length between 32.3 and 39.5 µm. The spermatozoa morphometry can be exploited to investigate relationships between sperm form and function under natural fertilization conditions (Snook, 2005). Difference in morphometry may represent a trade-off between speed of movement and longevity. Gage et al. (2002) found that sperm morphometry was related to a male’s sperm longevity for sperm total length. Sperm morphometry may also be related to the reproductive habit of the species. Gombar et al. (2017) found that *Oncorhynchus tshawytscha* males can have two distinct reproductive tactics. The larger and dominant males have priority in mating positions with females, while the smaller ones use the sneaking tactic during reproduction. Fish of this species with the sneaking tactic generally have superior sperm quality (Butts et al., 2012). In this sense, sperm morphometry represents a crucial factor for the reproductive success, and it is important to observe the variation in sperm size in a population of fish to identify possible variations in their reproductive habit. As there is little information about this in this species, characterization of sperm morphometry in *P. courbina* can serve as a reference to observe changes in sperm patterns and help to understand its reproductive strategy.

The LIVE/DEAD assay was used to show that the cells had a high membrane integrity (>70%), with results like those found in fresh sperm from Salvelinus fontinalis (Lahnsteiner, Mansour & Kunz, 2011). This guarantees that the cells presented good viability when performing the other analyses and we were sure that the reproductive cells were able to be analyzed when we applied the LIVE/DEAD validation test.

It is evident that the meticulous description of the morphological aspects of the spermatozoa, associated with the variables of sperm kinetics, fundamentally contributed to the development of an efficient reproductive protocol for *P. courbina* in the laboratory. With this, we understand that our findings are fundamental for future studies with this species, whether with a focus on an environmental conservation program (stock enhancement) or for the development of the species for aquaculture.

## Conclusions

Based on the observed results, it is possible to verify that the animals have sperm characteristics that indicate reproductive aptitude, such as high sperm VCL and morphometry similar to adult animals of other similar species. On the other hand, the high presence of the cytoplasmic gout abnormality in the spermatozoa indicates that the animals are not fully mature in their first reproductive season. The high frequency of abnormalities found may help to explain the unexpected behavior in sperm motility. The correlations found between sperm abnormalities and kinetics can help to understand this phenomenon, and the analysis of these parameters in fully mature animals would be the next step to be study in this species.

##  Supplemental Information

10.7717/peerj.15600/supp-1 Supplemental Information 1 Kinetics parameters data

10.7717/peerj.15600/supp-2 Supplemental Information 2 Morphometry, biometrics, membrane integrity analysis and morphological analysis data

10.7717/peerj.15600/supp-3 Supplemental Information 3 Methodology and graphical results of sperm parameters in P. courbina

10.7717/peerj.15600/supp-4 Supplemental Information 4 ARRIVE 2.0 Checklist

## Additional Information and Declarations

### Data Availability

The following information was supplied regarding data availability: The raw data is available in the Supplemental Files.
