# Supplementary figures and images for: Pogonias courbina sperm characteristcs in its first reproductive season
Source: PeerJ. 2023 Jul 18;11:e15600. doi: 10.7717/peerj.15600 (PMC10361073; doi:10.7717/peerj.15600)

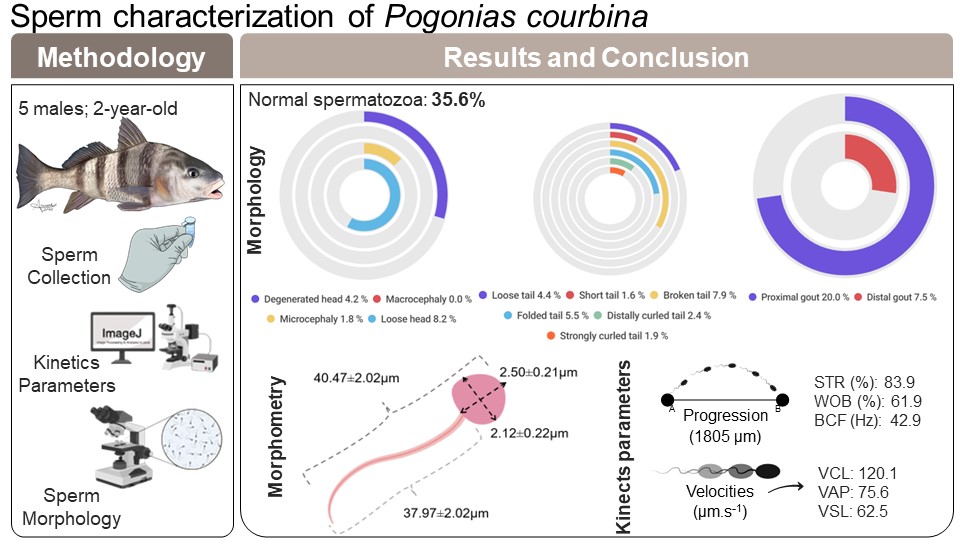

Supplement: Supplemental Information 3 [file peerj-11-15600-s003.jpg]
